# Wide Distribution and Diversity of Malaria-Related Haemosporidian Parasites (*Polychromophilus* spp.) in Bats and Their Ectoparasites in Eastern Europe

**DOI:** 10.3390/microorganisms9020230

**Published:** 2021-01-22

**Authors:** Attila D. Sándor, Áron Péter, Alexandra Corduneanu, Levente Barti, István Csősz, Zsuzsa Kalmár, Sándor Hornok, Jenő Kontschán, Andrei D. Mihalca

**Affiliations:** 1 Department of Parasitology and Parasitic Diseases, Faculty of Veterinary Medicine, University of Agricultural Sciences and Veterinary Medicine of Cluj-Napoca, RO-400036 Cluj Napoca, Romania; aronpeter92@gmail.com (Á.P.); alexandra.corduneanu@usamvcluj.ro (A.C.); bartilev@yahoo.com (L.B.); zsuzsa.kalmar@usamvcluj.ro (Z.K.); amihalca@usamvcluj.ro (A.D.M.); 2 Department of Parasitology and Zoology, University of Veterinary Medicine, H-1078 Budapest, Hungary; hornok.sandor@univet.hu; 3 Myotis Bat Conservation Group, RO-530171 Miercurea Ciuc, Romania; styepan@freemail.hu; 4 Centre for Agricultural Research, Plant Protection Institute, ELKH, H-1022 Budapest, Hungary; jkontschan@gmail.com

**Keywords:** Chiroptera, Ixodidae, Nycteribiidae, pathogens, Plasmodiidae

## Abstract

Malaria is responsible for major diseases of humans, while associated haemosporidians are important factors in regulating wildlife populations. *Polychromophilus*, a haemosporidian parasite of bats, is phylogenetically close to human-pathogenic *Plasmodium* species, and their study may provide further clues for understanding the evolutionary relationships between vertebrates and malarial parasites. Our aim was to investigate the distribution of *Polychromophilus* spp. in Eastern Europe and test the importance of host ecology and roost site on haemosporidian parasite infection of bats. We sampled bats and their ectoparasites at eight locations in Romania and Bulgaria. DNA was extracted from blood samples and ectoparasites and tested individually for the presence of DNA of *Polychromophilus* spp. using a nested PCR targeting a 705 bp fragment of *cyt*B. Two species of *Polychromophilus* were identified: *Po. melanipherus* in *Miniopterus schreibersii* and associated ectoparasites and *Po. murinus* in rhinolophid and vespertilionid bats (6 species) and their ticks and nycteribiid flies. Only cave-dwelling bat species (and their ectoparasites) showed infections, and we found a strong correlation between infections with *Polychromophilus* parasites and Nycteribiidae prevalence. We report the high genetic diversity of *Polychromophilus* spp. in Eastern Europe, suggesting that the simultaneous presence of varied host and vector assemblages enhances bat haemosporidian parasite diversity.

## 1. Introduction

Haemosporidians causing malaria are responsible for major diseases of humans (e.g., malarial infections in humans resulted in an estimated 228 million cases and 405,000 deaths in 2018 [1]). Certain species also act as important factors in regulating wildlife populations. The impact of malaria parasites was considered crucial in the extinction of Maclear’s rat (*Rattus macleari*) on Christmas Island [2], and it should be blamed for the extinction of up to 23 endemic Hawaiian bird species [3]. Recently avian malaria parasites were suggested to be the cause of the widespread decline of house sparrows (*Passer domesticus)* in Europe [4]. Host switching was recorded for several haemosporidian parasites [5,6], with even the most pathogenic human malaria species, *Plasmodium falciparum,* being suggested as a recent pathogen with a chimpanzee origin [7]. Other malaria-like haemosporidian parasites occurring in wildlife (e.g., *Polychromophilus* species of bats) are phylogenetically close to human-pathogenic *Plasmodium* species [8,9,10]; thus, their study may provide further clues in our fight against humanity’s most deadly infectious disease [11].

Bats are suggested or demonstrated reservoirs for a large variety of pathogens causing emerging infectious diseases such as viruses [12], bacteria [13], and protozoa [14,15,16]. The study of Hemosporidia [17] of bats can further provide an in-depth understanding of the evolutionary relationships between vertebrates and malarial parasites [6,18,19], especially in the view of the remarkable natural tolerance of bats towards these parasites [20,21].

Nine genera of Plasmodiidae infect bats worldwide: *Biguetiella*, *Bioccala*, *Dionisia*, *Hepatocystis*, *Johnsprentia*, *Nycteria*, *Plasmodium*, *Polychromophilus*, and *Sprattiella* [22] and based on current data; only *Polychromophilus* is present in Europe [6]. The genus *Polychromophilus* includes five species globally (*Po. adami*, *Po. corradetii*, *Po. deanei*, *Po. melanipherus* and *Po. murinus*), with two species infecting different hosts groups in Europe: *Po. melanipherus* is a parasite of the long-winged bat (*Miniopterus schreibersii*), while *Po. murinus* occurs mainly in vesper bats (Vespertilionidae) and certain rhinolophid species [23]. Unlike in other hosts, seemingly bats show little or no physiological symptoms associated with *Polychromophilus* infections [20].

The presence of *Polychromophilus* spp. are known in Europe from Great Britain, Italy, the Netherlands, and Switzerland [23,24,25,26]. However, to the best of our knowledge, there are no reports on malaria-related haemosporidian parasites of bats in the eastern part of the continent. As with most haemosporidians, *Polychromophilus* spp. are vector-borne parasites, with arthropod ectoparasites suggested to biologically transfer sporozoites between bats [27]. Bat flies (Diptera: Nycteribiidae) were suspected to be the main vectors of *Polychromophilus* spp. [27,28]. Thus, we hypothesize that bat species frequently infected by bat flies will show a higher prevalence of *Polychromophilus* spp. infections. In this context, our aims were to: (a) investigate the distribution of *Polychromophilus* spp. in Eastern Europe by sampling a diverse range of bat species, and (b) test whether there are differences in *Polychromophilus* spp. infection between primarily cave-dwelling (roosting in large underground shelters and commonly parasitized by bat flies) and crevice-roosting bat species (usually roosting in tree holes or crevices in built environments and rarely hosting bat flies). We predict that bat species resident in caves should show higher *Polychromophilus* spp. infection rates, than crevice-dwelling bat species. We also screened different ectoparasites collected from *Polychromophilus*-positive bats in order to establish their possible carrier role for haemosporidian parasites.

## 2. Materials and Methods

**Figure 1:**
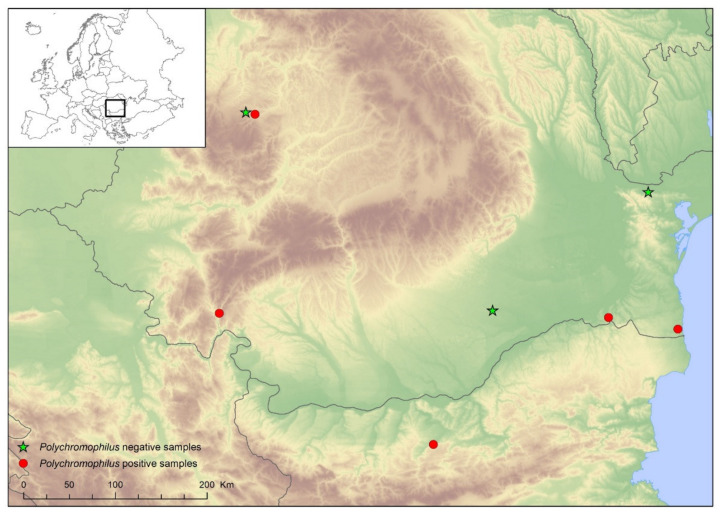
Geographical distribution of sampling locations used for testing *Polycromophilus* spp. presence in bats.

Blood samples were collected from live caught bats at eight different locations in Romania and Bulgaria (Figure 1, Table 1) in the spring and autumn of 2017 and 2018. For capturing the bats, mist nets and harp traps were set close to the entrances of roosts or in suitable habitat patches. Bats were identified morphologically [29], and species, sex, age, forearm length and body weight were recorded for each individual. Blood was collected from randomly allocated (using a preset list of random numbers generated by the RandBetween function of Excel), apparently healthy individuals using venipuncture. Each bat was immobilized, the uropatagium was disinfected with alcohol, and a puncture of the uropatagial vein was made using a small needle. The drop of blood was collected on a small piece of filter paper and kept in a sterile tube. Each tube was individually marked and stored at 4 °C until DNA extraction. Bat ectoparasites were also collected from bats and preserved in 70% ethanol in separate tubes (one tube/ectoparasite type/bat host). Identification of bat ectoparasites was based on morphological characteristics [30,31]. To assess the potential importance of host species ecology on *Polychromophilus* parasite prevalence, we assigned each bat species to one group (underground vs. crevice-roosting, see Table 1), according to published records [29].

Ectoparasites of bats with *Polychromophilus*-positive blood samples were selected and grouped in pools according to their species, host, developmental stage (for ticks only), and sex for DNA extraction. Ticks and bat flies were tested individually (24) or in pools (11 pools, 2–3 flies belonging to the same species and sex collected from the same host), with *Polychromophilus* spp. Genomic DNA was extracted from the blood from filter papers using an Isolate II Genomic DNA kit (Bioline, London, UK). The genomic DNA of bat ectoparasites was extracted using the QIAamp DNA Mini Kit (Qiagen, Hilden, Germany) according to the manufacturer’s instructions. All the DNA samples were stored at −20 °C until further analysis.

A nested PCR targeting a 705 bp fragment of *cyt*B gene using previously described primers [6] was used for screening. The reactions were carried out as follows: 25 µL reaction mixture containing 12.5 µL Master Mix (My Taq^TM^ Red Mix, Bioline, London, UK), 7.5 µL water, 1 µL of each primer (10 pmol/μL) and 3 µL aliquot of isolated DNA in the first round and in the second round instead of DNA 1 µL of PCR product from the first reaction was used. The PCR was performed using the T1000^TM^ thermal cycler (Bio-Rad, London, UK) with the following condition: initial denaturation at 94 °C for 5 min, then 25 cycles (for the first reaction) and 35 cycles (for the second reaction) of denaturation at 94 °C for 30 s, annealing at 47.2 °C for 30 s (for both reactions), and extension at 72 °C for 45 s and a final extension at 72 °C for 10 min. For each set of reactions (45 samples) 2 negative controls (distilled water) and one positive control, which was *Polychromophilus* spp.-positive DNA isolated from bat flies of Common bent-wing bat (*Mi. schreibersii*) collected from Italy were included.

Amplification products were visualized by electrophoresis on 1.5% agarose gel stained with RedSafe™ 20,000× nucleic acid staining solution (Chembio, Rickmansworth, UK), and their molecular weight was assessed by comparison to a molecular marker 100 bp DNA Ladder (O’GeneRuler ^TM^, Thermo Fisher Scientific, Waltham, MA, USA). PCR products were purified and sequenced (Macrogen Europe, Amsterdam, Netherlands). Obtained sequences were manually edited, then aligned and compared to those available in GenBank^TM^ by basic local alignments tool (BLAST) analysis. The MEGA model selection method was applied to choose the appropriate model for phylogenetic analyses. In the phylogenetic analyses, reference sequences with high coverage (i.e., 99–100% of the region amplified here) were retrieved from GenBank and trimmed to the same length. Phylogenetic analyses were conducted by MEGA version 7.0 using the maximum-likelihood method, Hasegawa–Kishino–Yano (HKY) model according to the selection of the program and 1000 bootstraps. The sequences were deposited in GenBank under the following accession numbers (*Po. melanipherus*: MT996236, MT996237, MT996238, MT996239, MT996240, MT996241, MT996242, MT996243; *Po. murinus*: MT996244, MT996245, MT996246, MT996247, MT996248).

## 3. Results

**Figure 2:**
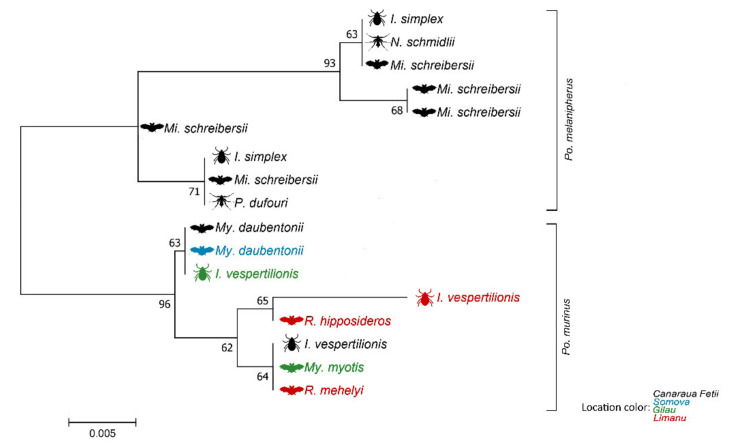
Un-rooted tree representing phylogenetic relationships between *Polycromophilus* spp. sequences collected from bats and their ectoparasites (Ixodidae and Nycteribiidae) in SE Europe. Color indicates sampling site, while symbols indicate organism type sampled (bats, bat flies and ticks). The scale-bar indicates the number of substitutions per site.

**Figure 3:**
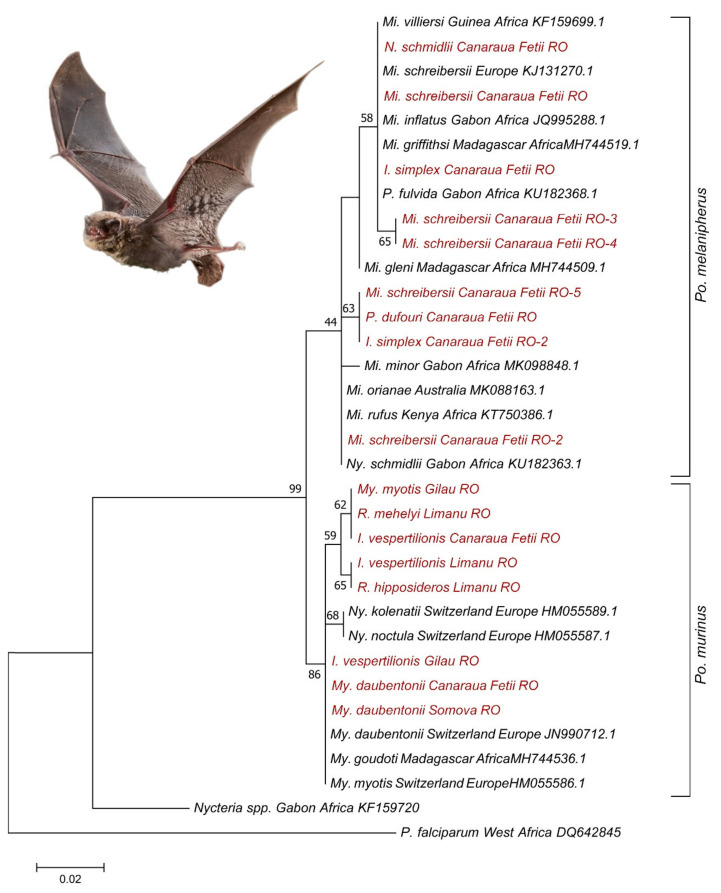
Phylogenetic comparison of *cyt*B sequences of *Polychromophilus* spp. The genotypes of Plasmodiidae sequences collected in this study are marked with red color. Branch lengths represent the number of substitutions per site inferred according to the scale shown.

Blood samples were taken from a total of 270 bats belonging to 19 species (Table 1, Figure 1). Among these bats, 59 individuals had ectoparasites, which were also included in the analysis. The DNA of *Polychromophilus* spp. was identified in the blood samples of 50 bats (general prevalence was 18.5%, CI: 14.3–23.5%), belonging to 7 species. In the case of positive samples, prevalence showed wide variations among different species, ranging from 10% (*Myotis blythii*) to 67.3% (*Mi. schreibersii*), or even 75% (*My. myotis*; see also Table 1). Sequencing showed the presence of two *Polychromophilus* spp. (Figure 2 and Figure 3). The samples from *Mi. schreibersii* showed a 99–100% identity to *Po. melanipherus* from Central and Southern Europe (Switzerland and Italy), but also showed an identity of 97.7–98.5% with *Po. melanipherus* collected from *Mi. gleni* in Madagascar. Blood samples from rhinolophid and vespertilionid bats all hosted different sequences of *Po. murinus*, with a sequence identity of 96.3–99.8% to reference sequences from bats (*My. daubentonii*, *My. myotis*) sampled in Switzerland and deposited in GenBank^TM^ (Figure 2).

Ectoparasites were collected at three locations (Băile Herculane, Canaraua Fetii and Limanu Cave, all in Romania). Two bat species were infested with ticks (prevalence 54.2%, mean intensity 4.2 tick/host, Table 2 and Table 3): *Mi. schreibersii* carried *Ixodes simplex* (prevalence 78.9%, mean intensity 4.4), while *My. daubentonii* was infected by *I. vespertilionis* (prevalence 11.8%, mean intensity 2.0, for other details, see Sándor et al. 2019 [32]). Bat flies (n = 53, seven species, mean prevalence 11.1%) were collected from five host species, among which the highest prevalence and diversity was recorded in the case of *Mi. schreibersii* (Table 2).

Altogether, 33 tick samples (10 individuals and 23 pools) were tested for Plasmodiidae DNA, and six *I. simplex* pools (8.9%) and three individuals of *I. vespertilionis* (one individual and one pool, 66.6%) were positive for *Polychromophilus* spp. (Table 2). Three of the positive pools of *I. simplex* contained larvae (2, 3, and 12, respectively), and further three consisted of three nymphs, collected from five different *Mi. schreibersii* individuals. The positive *I. vespertilionis* pool was made from two larvae. All these ectoparasites originated from bats that tested positive for *Polychromophilus* spp. However, only 20% of all DNA samples of ticks collected from *Polychromophilus-*positive bats were PCR-positive. The species identified with sequencing was *Po. melanipherus* (99.5–100% identity with KJ131274.1) in *I. simplex* pools, while *I. vespertilionis* harbored *Po. murinus* (99.2% identity with HM055588.1). Interestingly, corresponding sequences between ticks and their host individual did not show 100% sequence identity in each of the cases, with just a 94.9% identity between a 3 larva pool and the collecting host (32 linked single-nucleotide polymorphism—SNP difference, 23 deletions and 9 substitutions, 598/630 bp, see also Figure 2).

DNA of *Polychromophilus* spp. was detected in 23 fly samples (prevalence: 62.1%). Five different fly species contained the DNA of haemosporidians, with high prevalence rates recorded in *Penicillidia conspicua* (8/11, Table 3). No infection was found in *N. latreillii* (n = 3) and *N. pedicularia* (n = 3). Both species of *Polychromophilus* spp. were identified in bat flies. In particular, flies collected from *Mi. schreibersii* (*N. schmidlii, Pe. conspicua* and *Pe. dufourii*) contained the DNA of *Po. melanipherus*, while flies collected from vespertilionids (*N. kolenatii, N. vexata* and *Pe. dufourii*) all tested positive for *Po. murinus*. These are the first records of *Polychromophilus* spp. identified in nycteribiid flies in Eastern Europe (Romania), with first-ever records of *Po. melanipherus* in *Pe. conspicua* and *Pe. dufourii,* and the first-ever records of *Po. murinus* in *N. vexata* and *Pe. dufourii*. DNA of *Po. melanipherus* was found in *Pe. dufourii,* collected from *Mi. schreibersii* (Canarau Fetii), while individuals of the same dipteran species (collected from *My. blythii* and *My. myotis* at two different sites) tested positive for *Po. murinus*.

We found high sequence diversity of both *Polychromophilus* spp. identified (Figure 2). Altogether six different haplotype groups differing in at least 5 SNPs were identified among sequences belonging to *Po. melanipherus* (highest difference between two sequences was 5.3%, 38 SNP, 12 substitutions and 26 deletions, 626/664 bp), while four haplotype groups were identified among different sequences of *Po. murinus* (highest difference between two *Po. murinus* sequences was 3.9%, 25 base pairs, 8 substitutions and 17 deletions, 615/640 bp), while highest identity was 100% (700/702 bp). We found no geographical structuring in haplotype diversity. High identity sequences (99.99–100%) were found at geographically distant locations (e.g., Mandrata Cave, Somova and Gilău, ca. 400 km distance either direction), while single sites held high haplogroup diversity (in case of *Po. melanipherus*, all six haplogroups were located at Canaraua Fetii, while three out of four haplogroups of *Po. murinus* were identified at Limanu).

DNA of *Polychromophilus* spp. was identified only in cave-dwelling bat species (Table 1) and showed a strong correlation with Nycteribiidae prevalence on host species (Pearson Rank Correlation, R_(17)_ = 0.9406, *p* < 0.001). We found no statistically significant effect of neither bat sex nor age or bat fly sex on *Polychromophilus* spp. prevalence. Capture season had no effect on prevalence or haplotype diversity.

## 4. Discussion

Here we report on the occurrence of *Polychromophilus* spp. DNA in the blood of seven European insectivorous bat species and their tick and bat fly ectoparasites. These observations are the first geographical records of malaria-like bat parasites from Romania (both *Po. melanipherus* and *Po. murinus*) and Bulgaria (*Po. murinus*), thus further expanding the known host and geographic ranges of *Polychromophilus* spp. These results confirm the wide geographical distribution of both species of *Polychromophilus* in Europe after they were reported from the central, southern and western parts of the continent [23,24,25,26,33,34].

With regard to host specificity of malaria-like parasites in bats, in our study *Po. melanipherus* was reported only from the common bent-wing bat (*Mi. schreibersii*), while *Po. murinus* had a wider host range. *Polychromophilus melanipherus* is a fairly common parasite of the genus *Miniopterus* worldwide, with at least 20 different species of bent-winged bats recorded as hosts in Europe, Africa and Australia (Table S1). While other bat species (belonging to Hipposideridae, Pteropodidae and Vespertilionidae) were recorded as hosts of *Po. melanipherus* in Africa and Australia, all European records of *Po. melanipherus* relate to *Mi. schreibersii* (Table S1 and references therein).

*Polychromophilus murinus* is the type species of the genus [35], originally being described from the particolored bat (*Vespertilio murinus*) and later recorded in at least six other European bat species (*Myotis daubentonii, My. myotis, My. mystacinus, My. nattereri, Eptesicus serotinus*, and *Nyctalus noctula*) (Table S1). Here we report *Po. murinus* DNA in three new bat hosts, with the first records listed for *My. blythii, Rhinolophus hipposideros* and *R. mehelyi.* We also reconfirmed the presence of *Po. murinus* in three bat species (*My. daubentonii*, *My. myotis* and *R. ferrumequinum*) [19,23,27,36,37]. Five species were previously shown to harbor *Polychromophilus* spp. tested negative in our study. While several of these species were sampled in small numbers (n = 1–12), in the case of *N. noctula,* the sample size was relatively large (n = 134) from two different locations. Thus, our results indicate a low probability of infection for this bat species, at least in the investigated geographical territory.

A high prevalence of infection with *Polychromophilus* spp. was recorded in three bat species here, with values exceeding previous European records [19,23,27,36,37]. The mean prevalence for *Po. melanipherus* was 67.3% in *Mi. schreibersii,* while *Po. murinus* had a mean prevalence of 56.2% in the case of *My. daubentonii* and an even higher level (75%) in *My. myotis.* Moreover, these prevalence rates were recorded at more than one site, thus suggesting a widespread presence of *Polychromophilus* spp. in these bat species.

Only 20% of ticks collected from bats in this study were found to contain the DNA of either of the two *Polychromophilus* species, with 8 out of the 33 tick individuals/pools testing positive (Table 2). Both positive pools of *I. vespertilionis* came from Daubenton’s bat *(My. daubentonii*). However, the six positive pools of *I. simplex* (prevalence 19.3%, Table 2) came from several different hosts. While all these ticks were collected from hosts (thus probably consumed host–blood and may show host-derived *Polychromophilus* DNA), their vectorial role cannot be excluded and warrant further research. Interestingly, corresponding sequence pairs (ticks and their respective bat–host individual) did not show 100% identity in two of the cases recorded in this study (Figure 2). Either more than one *Polychromophilus* spp. geno-sequence was present in the sampled individuals (with nPCR being able to identify only one, an expected caveat of the methodology [38]) or ticks still maintained *Polychromophilus* DNA fragments from a previous meal (and host, in case of nymphs), not an unusual property of ixodid ticks [39]. To establish the vectorial role of ectoparasites, one should prove that haemosporidian parasites are able to finish their cycle inside the ectoparasite individual [40].

Altogether five different bat fly species hosted haemosporidian DNA, and one of them (*Pe. dufourii*) tested positive for both *Polychromophilus* species. Here we report the first-ever records of *Po. melanipherus* in *Pe. conspicua* and *Pe. dufourii* and the first-ever records of *Po. murinus* in *N. vexata* and *Pe. dufourii.* Nycteribiid bat flies were proposed to be the main vectors of *Polychromophilus* spp. in bats [27,36]. While no experimental proof has yet been published, their ubiquitous presence on bats, coupled with the high prevalence of *Polychromophilus* spp. recorded in bat flies themselves [20,33,34] suggest this. Our results indirectly support this hypothesis. We identified a significantly higher prevalence of *Polychromophilus* DNA in cave-dwelling bat species known to regularly host bat flies in high abundance in Romania (*Mi. schreibersii, My. blythii, My. daubentonii* or *My. myotis* see [41]). Bat species with low levels of fly parasitism or rarely hosting nycteribiids (e.g., *R. ferrumequinum*) had low *Polychromophilus* DNA prevalence or tested negative for this parasite (*E. serotinus, My. emarginatus*, *N. noctula*, *V. murinus,* [41]).

Nycteribiid-related *Po. melanipherus* sequences clustered with bat-related *Po. melanipherus* sequences from Central and Southern Europe (99.3–100% identity, [33]) and from Madagascar (99.3% identity, [28]). *Polychromophilus murinus* sequences from bat flies were similar (92.4–98.7% identity) to samples collected from vespertilionid bats in Switzerland (see also Figure 3).

*Polychromophilus melanipherus* showed high haplotype diversity both in bats and their ectoparasites. Two genetically different haplotypes were identified in the two bat fly species collected from bent-winged bats, while *I. simplex* ticks also provided different haplotypes. Unfortunately, our method prevented us from evaluating the true diversity of *Polychromophilus* spp. haplotypes in individuals sampled (both bats and/or ectoparasite individuals), as the method deployed (nested PCR) amplified only a single sequence from each sample. The six haplogroups identified clustered into two main groups, including all ectoparasite-derived and bat-derived sequences. While all *Po. melanipherus-*positive bats and ectoparasites were collected at a single site (Canaraua Fetii), this site hosts a huge maternity cluster of *Mi. schreibersii*, with up to 8000 individuals in summer [42,43], but no bats present in winter. Local recapture of ringed bats suggests multiple wintering areas for these bats, with hibernacula known in the NE, E, SE and SW ([44] and Barti L. unpublished). These subpopulations may host different genetic lineages of *Po. melanipherus*, all of which may be encountered at the maternity roost at Canaraua Fetii.

The high diversity of individual *Polychromophilus* spp. sequences encountered in bats, ticks, and bat flies (see Figure 2) suggest an intricate web of malaria-like parasite circulation among bats and their ectoparasites in eastern Europe, with multiple genotypes present even at the site level. This is likely the result of (i) either the adaptation of individual genotypes to specific vector or host species (in the case of the multi-host *Po. murinus*) or (ii) the presence of multiple genetic lineages of *Po. melanipherus* linked to different migratory subpopulations of bent-winged bats.

DNA of both haemosporidian species was identified in a polyxenous bat fly species, *Pe. dufourii*, thus suggesting vector competence for both *Polychromophilus* species. This dipteran species commonly occurs on a number of cave-dwelling bat species like *Mi. schreibersii*, *My. blythii*, *My. daubentonii* or *My. myotis* [41], i.e., bat species of which populations may share the same underground roost (like in our case, the Canaraua Fetii site). At such roosts, individual bat flies may easily move not only between host individuals of the same species but also between different host species [45,46,47]. Thus, these flies have the potential to transfer both haemosporidian species to nonspecific bat hosts (e.g., *Po. melanipherus* to vesper bats and/or *Po. murinus* to *Mi. schreibersii*), too. The fact that we found no sign of such cross-infection in any of the host species may be an indication that there is a specific barrier of infectivity at the host level, an idea already suggested for *Po. melanipherus* [28].

## 5. Conclusions

Our results expanded the known geographical range of bat-associated Plasmodiidae species occurring in the Western Palearctic, showing wide distribution among bats and their ectoparasites in SE Europe. Here we report the first records of *Polychromophilus murinus* in three new bat species and *Polychromophilus* spp. in two tick and three bat fly species, thus increasing both host and possible vector species spectra. High genetic diversity is reported for both *Polychromophilus* species, with diverse genetic variants present even at the same location, suggesting that simultaneous presence of diverse host and vector assemblages may enhance malaria-like parasite diversity, too.

## Figures and Tables

**Table 1:** Species and numbers of bats sampled, with roost-type, geographical locations and sample sizes (Roost type: C—crevice roosting, U—underground shelters. Sampling locations: A—Bucharest; B—Canaraua Fetii; C—Gilău; D—Căpușu Mic; E—Limanu Cave; F—Mandrata Cave (Bulgaria); G—Băile Herculane; H—Telita).

Bat Species	Roost Type	Locations								Total	No Positive	*Polychromophilus*
		A	B	C	D	E	F	G	H		(%)	spp.
*Eptesicus serotinus*	C		1							1		
*Miniopterus schreibersii*	U		46							46	31 (67.4)	*Po. melanipherus*
*Myotis alcathoe*	C				4					4		
*Myotis blythii*	U		6	3				1		10	1 (10.0)	*Po. murinus*
*Myotis capaccinii*	U							1		1		
*Myotis daubentonii*	U		1			15				16	9 (56.2)	*Po. murinus*
*Myotis emarginatus*	C			10						10		
*Myotis myotis*	U			1			5	2		8	6 (75)	*Po. murinus*
*Myotis nattereri*	C		12							12		
*Nyctalus lasiopterus*	C		1							1		
*Nyctalus noctula*	C	95	39							134		
*Pipistrellus kuhlii*	C	2								2		
*Pipistrellus nathusii*	C		5							5		
*Plecotus austriacus*	C								1	1		
*Rhinolophus ferrumequinum*	U			9						9	1 (11.11)	*Po. murinus*
*Rhinolophus hipposideros*	U					2				2	1 (50)	*Po. murinus*
*Rhinolophus mehelyi*	U					2				2	1 (50)	*Po. murinus*
*Vespertilio murinus*	C	1	4							5		
Total		98	115	23	4	19	5	5	1	270		

**Table 2:** Ticks found on bats, with host species, parasite life stages and presence of *Polychromophilus* spp. DNA in ticks.

Host Species	No Samples (Infested)	*I. simplex*			*I. vespertilionis*			Total	*Polychromophilus* spp. Positive
		F	N	L	F	N	L		
*Miniopterus schreibersii*	30 (6)	1	48	37	-	-	-	86	16 L, 10 N
*Myotis daubentonii*	3 (2)	-	-	-	-	1	3	4	2 L, 1 N
Total	33 (8)	1	48	37	-	1	3	90	29 (29.2%)

F—female, N—nymph, L—larva.

**Table 3:** Bat flies (Nycteribiidae) analyzed for *Polychromophilus* spp. infection, with host species and haemosporidian species recorded.

Nycteribiidae/Host Species	Bat Fly Sex	*Miniopterus schreibersii*	*Myotis blythii*	*Myotis capaccinii*	*Myotis daubentonii*	*Myotis myotis*	No. of Positive Pools (Detected Species)
*Nycteribia kolenatii*	F				1		1 (*Po. murinus*)
	M						-
*Nycteribia latreillii*	F		2				-
	M		1				-
*Nycteribia pedicularia*	F			1			-
	M			2			-
*Nycteribia schmidlii*	F	9					4 (*Po. melanipherus*)
	M	16					3 (*Po. melanipherus*)
*Nycteribia vexata*	F						-
	M		1				1 (*Po. murinus*)
*Penicillidia conspicua*	F	7					4 (*Po. melanipherus*)
	M	4					3 (*Po. melanipherus*)
*Penicillidia dufourii*	F			1		2	2 (*Po. murinus*)
	M	3	2	1		3	1 (*Po. melanipherus*), 4 (*Po. murinus*)
Total		39	6	5	1	5	

## Data Availability

All data are contained within the article and the supplementary material.

## Supplementary Materials

The following are available online at https://www.mdpi.com/2076-2607/9/2/230/s1. Table S1: List of bat and ectoparasite species recorded to be harboring *Polychromophilus* spp., with geographical location and species of Plasmodiidae hosted.
